# The Role of [^18^F]Fluciclovine PET/CT in the Characterization of High-Risk Primary Prostate Cancer: Comparison with [^11^C]Choline PET/CT and Histopathological Analysis

**DOI:** 10.3390/cancers13071575

**Published:** 2021-03-29

**Authors:** Lucia Zanoni, Riccardo Mei, Lorenzo Bianchi, Francesca Giunchi, Lorenzo Maltoni, Cristian Vincenzo Pultrone, Cristina Nanni, Irene Bossert, Antonella Matti, Riccardo Schiavina, Michelangelo Fiorentino, Cristina Fonti, Filippo Lodi, Antonietta D’Errico, Eugenio Brunocilla, Stefano Fanti

**Affiliations:** 1 Nuclear Medicine Unit, Istituto di Ricovero e Cure a Carattere Scientifico (IRCCS), Azienda Ospedaliero-Universitaria di Bologna, 40138 Bologna, Italy; cristina.nanni@aosp.bo.it (C.N.); filippo.lodi@aosp.bo.it (F.L.); stefano.fanti@aosp.bo.it (S.F.); 2 Department of Experimental, Diagnostic and Specialty Medicine, Alma Mater Studiorum, University of Bologna, 40138 Bologna, Italy; riccardo.mei@studio.unibo.it (R.M.); michelangelo.fiorentino@unibo.it (M.F.); 3 Division of Urology, Istituto di Ricovero e Cure a Carattere Scientifico (IRCCS), Azienda Ospedaliero-Universitaria di Bologna, 40138 Bologna, Italy; lorenzo.bianchi3@gmail.com (L.B.); cristianvincenzo.pultrone@aosp.bo.it (C.V.P.); riccardo.schiavina3@unibo.it (R.S.); eugenio.brunocilla@unibo.it (E.B.); 4 Alma Mater Studiorum, University of Bologna, 40138 Bologna, Italy; lorenzo.maltoni2@studio.unibo.it; 5 Pathology, Istituto di Ricovero e Cure a Carattere Scientifico (IRCCS), Azienda Ospedaliero-Universitaria di Bologna, 40138 Bologna, Italy; francesca.giunchi@aosp.bo.it (F.G.); antonietta.derrico@aosp.bo.it (A.D.); 6 Nuclear Medicine, Istituti Clinici Scientifici Maugeri, 27100 Pavia, Italy; irene.bossert@icsmaugeri.it; 7 Nuclear Medicine, Istituto di Ricovero e Cure a Carattere Scientifico (IRCCS), Ospedale Sacro Cuore-Don Calabria, 37024 Negrar di Valpolicella (VR), Italy; antonella.matti@sacrocuore.it; 8 Istituto di Ricovero e Cure a Carattere Scientifico (IRCCS), Istituto delle Scienze Neurologiche di Bologna, 40139 Bologna, Italy; cristina.fonti@gmail.com

**Keywords:** [^18^F]Fluciclovine PET/CT, primary prostate cancer, high risk, staging, [^11^C]Choline

## Abstract

**Simple Summary:**

The role of [^18^F]Fluciclovine Positron Emission Tomography/Computed Tomography (PET/CT) in the characterization of intra-prostatic lesions was evaluated in high-risk primary PCa patients, scheduled for radical surgery, comparing investigational [^18^F]Fluciclovine and conventional [^11^C]Choline PET/CT results with the reference standard of pathologic surgical specimen. PET visual and semi-quantitative analyses were performed: for instance, patient-based, blinded to histopathology; subsequently lesion-based, unblinded, according to a pathology reference mapping. Among 19 pts, 45 malignant and 31 benign lesions were found. The highest SUVmax matched with the lobe of the index lesion in 89% of pts and a direct correlation between [^18^F]Fluciclovine uptake values and pISUP was demonstrated. Overall, the lesion-based performance of PET semiquantitative parameters (SUVmax, Target to background Ratio-TBRs) with either [^18^F]Fluciclovine or [^11^C]Choline, in detecting either malignant/ISUP2-5/ISUP4-5 PCa lesions, was moderate and similar (AUCs ≥ 0.70), but still inadequate (AUCs ≤ 0.81) as standalone staging procedure. TBRs (especially with threshold higher than bone marrow) may be complementary to implement malignancy targeting.

**Abstract:**

The primary aim of the study was to evaluate the role of [^18^F]Fluciclovine PET/CT in the characterization of intra-prostatic lesions in high-risk primary PCa patients eligible for radical prostatectomy, in comparison with conventional [^11^C]Choline PET/CT and validated by prostatectomy pathologic examination. Secondary aims were to determine the performance of PET semi-quantitative parameters (SUVmax; target-to-background ratios [TBRs], using abdominal aorta, bone marrow and liver as backgrounds) for malignant lesion detection (and best cut-off values) and to search predictive factors of malignancy. A six sextants prostate template was created and used by PET readers and pathologists for data comparison and validation. PET visual and semi-quantitative analyses were performed: for instance, patient-based, blinded to histopathology; subsequently lesion-based, un-blinded, according to the pathology reference template. Among 19 patients included (mean age 63 years, 89% high and 11% very-high-risk, mean PSA 9.15 ng/mL), 45 malignant and 31 benign lesions were found and 19 healthy areas were selected (*n =* 95). For both tracers, the location of the “blinded” prostate SUVmax matched with the lobe of the lesion with the highest pGS in 17/19 cases (89%). There was direct correlation between [^18^F]Fluciclovine uptake values and pISUP. Overall, lesion-based (*n =* 95), the performance of PET semiquantitative parameters, with either [^18^F]Fluciclovine or [^11^C]Choline, in detecting either malignant/ISUP2-5/ISUP4-5 PCa lesions, was moderate and similar (AUCs ≥ 0.70) but still inadequate (AUCs ≤ 0.81) as a standalone staging procedure. A [^18^F]Fluciclovine TBR-L3 ≥ 1.5 would depict a clinical significant lesion with a sensitivity and specificity of 85% and 68% respectively; whereas a SUVmax cut-off value of 4 would be able to identify a ISUP 4-5 lesion in all cases (sensitivity 100%), although with low specificity (52%). TBRs (especially with threshold significantly higher than aorta and slightly higher than bone marrow), may be complementary to implement malignancy targeting.

## 1. Introduction

Prostate cancer (PCa) is one of the most common tumors, with an increasing incidence in elderly men. A reliable detection and delineation of malignant lesions within the prostate is of utmost importance for risk-stratification and for tailored treatment planning [1,2,3]. Despite that PCa is a multifocal disease, there is evidence that the most aggressive lesion within the gland may play a significant prognostic role being responsible for the metastatic and recurrent disease [4].

To date, diagnostic sensitivity and risk-stratification is limited by sampling errors and by suboptimal imaging performance and availability.

Multi-parametric Magnetic Resonance Imaging (mpMRI) represents the most useful method for local staging, being able to depict size and extension of disease to adjacent organ and to improve the surgical planning, despite still not sensitive for microscopic extra-prostatic-extension (EPE) [5,6]. The use of high field strength (3 Tesla) or functional imaging improves sensitivity and, when combined with clinical data, increases pathological stage prediction [1]. A recent meta-analysis demonstrated that mpMRI had a pooled sensitivity of 0.91 (95% CI: 0.83–0.95) and a pooled specificity of 0.37 (95% CI: 0.29–0.46) for International Society of Urological Pathology (ISUP) grade >2 cancers [7], whereas for ISUP grade >3, 0.95 (95% CI: 0.87–0.99) and 0.35 (95% CI: 0.26–0.46), respectively. However, MRI still have some limitations: it detects less than 30% of ISUP grade 1 smaller than 0.5 cc and has poor sensitivity for central prostatic gland involvement; it suffers from significant false-positive (FP) rates in benign prostatic hyperplasia (BPH); despite the use of standardized Prostate Imaging—Reporting and Data System (PIRADSv2) score [8], mpMRI inter-reader reproducibility remains mild and requires dedicated radiologists.

Positron Emission Tomography (PET) is a whole-body non-invasive imaging modality that has great diagnostic value providing information about functional metabolic activity or receptor expression, not available with other conventional techniques [9].

Positron Emission Tomography/Computed Tomography (PET/CT) performed with the standard tracer [^11^C]Choline has the short [^11^C] half-life (T/2 = 20 min) as main disadvantage, limiting its use only to cyclotron-based PET centers [10]. On the contrary, the choline labelled with [^18^F] (longer T/2 = 120 min) can be transported to centers without cyclotrons onsite, but it is affected by earlier and higher physiological urinary excretion, hampering prostatic bed exploration and provides comparable suboptimal diagnostic sensitivity, due to the generally slow proliferating index of PCa cells that may reduce choline uptake. In addition, reflecting increased choline transport and overexpression of choline kinase, not only PCa but also BPH and prostatitis may cause high tracer accumulation [11]. As a significant uptake overlap between malignant and benign lesions exists, decreasing tracer specificity, the clinical utility of Choline PET/CT scan in primary PCa remains controversial.

On the other hand, more recently, Prostate specific membrane antigen (PSMA)-based PET/CT revealed more favorable diagnostic performance (see Section 4.4) [12,13].

More than a decade ago, Mark Goodman’s laboratory at Emory University developed a non-natural amino acid, Anti-1-amino-3-[^18^F]fluorocyclobutane-1-carboxylic acid, or [^18^F]FACBC, which is taken up into cells via the sodium-independent L-type amino acid transporter (LAT1) and the sodium-dependent neutral amino acid transporter (ASCT2), up-regulated in many human cancers, including PCa. This new metabolic tracer demonstrated several favorable characteristics: low accumulation in brain and pelvic region, low and homogeneous uptake in normal tissue backgrounds, high in vivo stability and slow urinary excretion which may be associated with a non-metabolized nature; relatively short synthesis time, long half-life (T/2 = 109 min) being [^18^F]-labelled [14,15,16,17]. Several trials demonstrated its clinical potential in both primary [18] and recurrent PCa [19,20,21,22,23,24,25], in particular, based on data collected from 797 PCa patients, it has been recently (2017) registered as [^18^F]Fluciclovine (Axumin; Blue Earth Diagnostics, Inc, Oxford, GB), authorized by Food and Drug Administration (FDA) and European Medicines Agency (EMA) for PET imaging in adult men with a suspected recurrence based on elevated blood prostate specific antigen (PSA) levels after primary curative treatment [24,26,27]. Due to radiopharmaceutical developments, an increased use also in different settings from biochemical relapse (BCR), i.e., in primary PCa, may be anticipated. At the time when the present study-design was conceived, a few previous studies already demonstrated fast [^18^F]Fluciclovine uptake and slow wash-out in the prostate tumors [14,17,28,29], but also documented potential reasons for FP, including BPH, post-radiation inflammation and fibrosis [18]. Schuster and colleagues [18] retrospectively demonstrated that visual analysis of [^18^F]Fluciclovine uptake is significantly correlated to disease either at prostate gland and pelvic nodes in 15 patients with intermediate/high-risk PCa, showing 80% sensitivity and 50% specificity. Moreover, [^18^F]Fluciclovine SUV max was found directly and statistically correlated to Gleason Score (GS), suggesting a potential role in detecting the most aggressive areas and thus, leading prostatic biopsy. Turkbey and collaborators [28] found the kinetic activity peak at 3.6 min post-injection (p.i.) and a relative plateau at 15–20 min (min) in a series of 21 PCa patients (pts); despite good sensitivity (90%) for dominant lesion detection, malignant zone uptake was higher than healthy one but indistinguishable from BPH; however, combined [^18^F]Fluciclovine PET/CT and T2-weighted MR imaging enabled more accurate PCa lesions’ localization (positive predictive value-PPV = 80%) than either modality alone (sector-based sensitivity and specificity: 67% and 66% for [^18^F]Fluciclovine and 73% and 79% for MRI, respectively).

There is growing interest in the relationship between PET uptake parameters and biologic behavior of primary PCa; however, a deep knowledge about tracer accumulation in different prostatic tissues would be the first and main step towards this purpose. Validation of new methods for non-invasive detection of PCa aggressiveness could enable improved risk-stratification.

With the present study we aimed to investigate the role of [^18^F]Fluciclovine PET/CT in the characterization of intra-prostatic lesions in men with high-risk primary PCa scheduled for radical prostatectomy (RP) and pelvic lymph-node dissection (PLND), comparing investigational [^18^F]Fluciclovine and conventional [^11^C]Choline PET/CT results with the reference standard of pathologic surgical specimen.

## 2. Materials and Methods

### 2.1. Primary and Secondary Aims

The primary aim of the study was to evaluate the role of [^18^F]Fluciclovine PET/CT in the characterization of intra-prostatic lesions in high-risk primary PCa patients eligible for RP, in comparison with conventional [^11^C]Choline PET/CT and validated by prostatectomy pathologic examination.

Secondary aims were to determine the usefulness of PET semi-quantitative parameters for malignant lesion detection; to investigate the best cut-offs value of PET parameters for malignant lesion detection; to identify clinical/imaging predictive factors of malignancy.

### 2.2. Study Design

This work is part of a prospective monocentric study (“^18^F-FACBC for staging high risk prostate cancer”; Local Ethical Committee code: 139/2014/O/Sper; Eudract number 2014-003165-15), which enrolled, between January 2015 and December 2016, a consecutive series of intermediate-to high-risk PCa patients who were scheduled for RP and PLND. All subjects signed a written informed consent form before undergoing the investigational [^18^F]Fluciclovine PET/CT procedure, additional to the standard staging work-up. The results of [^18^F]Fluciclovine imaging were compared with the data deriving from the other biochemical, clinical and instrumental investigations performed within the normal care pathway. Histopathological examination of surgical specimens was considered as the Standard of Truth for data validation.

Among patients who underwent [^18^F]Fluciclovine PET/CT (*n =* 94), all the following inclusion criteria were considered for the specific purpose of the present analyses.

#### 2.2.1. Inclusion Criteria

Patients with newly diagnosed, biopsy confirmed, high-risk PCa as defined by EAU Guidelines [1] (cT2c, cT3a or Gleason score 8–10 or PSA > 20 ng/mL); who underwent conventional staging [^11^C]Choline PET/CT, less than 1 month before, excluding definite bone metastases and who were addressed to RP with PLND within 3 months from [^18^F]Fluciclovine scan; presenting with all standard pre-operative staging investigations at our Institute (IRCCS, Azienda Ospedaliero-Universitaria-AOU di Bologna), including an accurate histopathological analysis of prostatectomy, specifically identifying the precise location, nature and type of each malignant and benign intraprostatic lesion.

A workflow diagram of the study design is presented as Supplementary Material (Figure S1).

#### 2.2.2. Exclusion Criteria

Patients presenting with at least one of the following criteria were excluded: age < 18 years; unable to undergo [^18^F]Fluciclovine PET/CT scanning for any reason; affected by severe acute co-morbidity/contraindications to surgery, e.g., debilitating cardiac, pulmonary, or neurologic disease; presenting with definite bone metastases at pre-operative imaging; being diagnosed with intermediate-risk primary PCa; who performed conventional [^11^C]Choline PET/CT more than 1 month before [^18^F]Fluciclovine PET/CT and/or underwent prostatectomy after more than 3 months from [^18^F]Fluciclovine PET/CT; receiving pre-operative specific oncologic therapy (i.e., hormonal therapy, chemotherapy, radiotherapy).

In particular, the short time interval between the two PET scans and between imaging and surgery and the absence of neo-adjuvant hormonal therapy was defined in order to potentially avoid any significant change and evolution of the clinical and pathological intra-prostatic disease status and for a better results comparison between the different procedures.

### 2.3. Radiotracer Production

In this study, the radiopharmaceuticals [^11^C]Choline (used in normal clinical practice) and [^18^F]Fluciclovine (investigational at the time of the study period) were both synthetized within the Radiopharmacy Laboratory of the Nuclear Medicine Unit (IRCCS, AOU di Bologna). A General Electric PETtrace 16.5 MeV cyclotron was employed.

The synthesis and quality control of [^11^C]Choline was performed in compliance with the guidelines of the European Pharmacopoeia [30].

At the time of the study enrolment, [^18^F]Fluciclovine was investigational: it was produced basing on cyclotron produced [^18^F] (necessary for the tracer labelling) and a synthesis module pre-loaded with single use cassettes, according to a previously reported method developed at Emory University [20]; dosimetry estimates fell within the acceptable limits of radiation exposure (Radioactive Drug Research Committee), similar to or lower than those of clinically approved radiotracers, including [^18^F] fluorodeoxyglucose (FDG). More recently the tracer has been authorized (AIC AIFA number: 045431) [27].

### 2.4. PET/CT Protocol

Conventional [^11^C]Choline PET/CT was performed following national and international guidelines (approximately 370 MBq intravenous-i.v.-administration, uptake time approximately 5 min, PET acquisition 2 min/bed position) [31].

Concerning investigational [^18^F]Fluciclovine, the patient was recommended to fast for at least four-hours (except for sips of water) and to avoid any significant physical exercise from the previous day. Approximately 370 MBq ± 20% was injected intravenously, with a slow injection followed by a flushing of physiological solution (max 10 mL) to patient with arms turned downwards, possibly in the right arm. After injection and washing, the patient returned his arms to the scanning position. The uptake time was approximately 4 min (range 3–5 min).

The images were acquired in a supine position with a 3D PET/CT Tomograph (Discovery 710, GE Medical Systems, Milwaukee, WI, USA) for 3 min for bed position for the emission phase. Field of view (FOV) was extended from proximal femur to the skull vertex. Low-dose CT scan (120 kV, 80 mA), with no oral nor i.v. contrast agent, was performed for attenuation correction and anatomical mapping. Emission data were corrected for scatter, random events and dead time of the system using specific software.

### 2.5. Imaging Interpretation

At least two nuclear medicine physicians with extensive experience in oncology imaging evaluated the PET/CT scans. When there was a disagreement, a third reader was involved for the final consensus. A dedicated workstation was used (Advantage ADW, GE Healthcare WI, USA) with a simultaneous visualization system of PET, CT and fused PET/CT images in axial, sagittal and coronal views.

A six sextants-prostate template was created and used independently by PET readers and pathologists for anatomical mapping, data comparison and validation.

PET interpretation was both visual and semi-quantitative.

All clinical, imaging and pathological data, relevant for final clinical staging, were collected.

#### 2.5.1. Patient-Based Interpretation

For instance, PET scans were read with full knowledge of the pre-operative clinical data but blinded to prostatectomy pathologic results. PET visual positivity criteria was presence of areas of increased tracer uptake above the surrounding prostate background and outside areas of physiological uptake, in the presence or absence of an identifiable lesion at the corresponding low-dose CT. Uptake pattern was defined as faint/mild/moderate/severe, focal/diffuse, homogeneous/heterogeneous.

The following PET/CT semi-quantitative parameters were measured: maximum Standardized uptake value (SUVmax) of the right and left prostate lobe; metabolic tumor volume (MTV; tracer avid volume that presents a threshold >42% of the max SUV, expressed in cm^3^) of the whole prostate.

#### 2.5.2. Lesion-Based Interpretation

PET/CT images with both radiopharmaceuticals, subsequently, were retrospectively evaluated following the result of the histological analysis performed on the excised gland (unblinded imaging revision according to the reference prostate template). Semi-quantitative analysis was extended, measuring lesion-SUVmax positioning, for each prostate gland, different volumes of interest (VOIs) in correspondence with each benign and malignant lesion highlighted by the pathologist template and a 1 cm^3^-VOI in a selected healthy zone. Furthermore, Target-to-background ratios (TBRs) were calculated, given by the ratio between each lesion-SUVmax and the average SUV (SUVmean) at the level of reference backgrounds, located, respectively, in:

- abdominal aorta (1 cm^3^-VOI within the vessel lumen), called TBR-AORTA;
- bone marrow (1 cm^3^-VOI at L3 vertebral body), called TBR-L3;
- liver (3 cm^3^-VOI in healthy hepatic parenchyma, when possible in the right lobe), called TBR-LIVER.

### 2.6. Histopathological Analysis

The surgical plan consisted of RP with PLND with open, laparoscopic or robotic approach at the Division of Urology (IRCCS, AOU di Bologna) by experienced surgeons, as previously described [32,33], following standard recommendations [1], decided according to clinical and conventional imaging staging work-up and not altered by the results of the investigational scan.

The histo-pathological examinations were all performed by Pathological Anatomy Unit (IRCCS, AOU di Bologna), as part of standard practice and in accordance with European guidelines [1,2,3]. At least one experienced, dedicated uro-pathologist (F.G.) reviewed all cases. The referring pathologist divided the prostate gland into six different sextants: base, mid/intermediate, apex (left and right). For each sextant the presence of malignant lesion, the specific histotype and the corresponding pGS was recorded; furthermore, the number of positive nodules divided per right and left lobe and pathological staging (pTNM) were evaluated. The index lesion was identified as the lesion with the highest pGS. Furthermore, malignant lesions were classified according to pISUP grade, in particular: clinically significant lesion (pISUP 2–5) and high-grade PCa (pISUP4–5). The tumor margins were delineated manually. In addition, the slices were screened for benign lesions: prostatic intraepithelial neoplasia (PIN), BPH and inflammation (prostatitis), post-atrophic hyperplasia (PAH) and infarction. For better anatomical localization, malignant and benign lesions were manually mapped into the reference prostate template.

### 2.7. Data Comparison and Validation

The rationale for this approach was the comparison of the investigational [^18^F]Fluciclovine imaging with standard clinical practice. All the conventional imaging scans were scheduled clinically and reimbursed as part of their standard clinical care and medical record. [^18^F]Fluciclovine PET/CT imaging results were compared with the reference standard of histological findings in surgical specimen and were also evaluated in comparison to the conventional tracer available at our Center, [^11^C]Choline.

### 2.8. Statistical Analyses

Means, standard deviations, medians, range, interquartile range (IQR) and frequencies were used as descriptive statistics.

#### 2.8.1. Patient-Based Analyses

Radiotracer distribution (SUVmax) and index lesion location (in terms of laterality- right, left or bilateral prostate lobes- not per sextant) were visually compared. We assessed whether the location of intraprostatic SUVmax corresponded to the location of the index malignant lesion (with the highest pGS) subsequently identified at histology. Metabolic Tumor Voume (MTV) of the whole prostate gland were also calculated and compared.

#### 2.8.2. Lesion-Based Analyses

PET/CT semi-quantitative data (SUVmax and TBRs) were correlated to pISUP through a lesion-based linear correlation (Spearman). Linear coefficients were estimated. Benign and healthy tissue areas were grouped as “non-malignant” when clinically relevant for the analyses. Wilcoxon–Mann–Whitney and Kruskal–Wallis tests were employed to investigate the capability of both tracers to differentiate between malignant and non-malignant lesions or between malignant, benign tissue and healthy tissue, respectively.

For both imaging modalities ([^18^F]Fluciclovine and [^11^C]Choline PET/CT) Receiver operating characteristic (ROC) curves of each semiquantitative parameter (SUVmax, TBRs) were calculated to evaluate the diagnostic performance for malignant lesion detection, clinical significant lesion (pISUP 2–5) and for high-grade PCa (pISUP 4–5). Area Under the curves (AUCs) with the 95% confidence intervals (C.I.) are reported and compared to each other as well as to a fixed value of 0.5. We also identified the best cut-off values (Youden Index) to discriminate either a malignant lesion or a clinically significant (ISUP 2–5) or a high-grade (ISUP 4–5) lesion.

Furthermore, univariate and multivariate logistic regression were performed to predict PCa malignancy at final pathology including followings covariates: iPSA, clinical stage, clinical ISUP, PIRADS v.2, [^11^C]Choline and [^18^F]Fluciclovine PET/CT parameters (SUVmax, TBRs).

All statistical tests were performed using SPSS 25 for Windows. A *p* < 0.05 was considered the threshold for statistical significance.

## 3. Results

### 3.1. Patient-Based Analyses (Blinded to Histopathology)

Overall, 19 patients were included in the final analysis. The tracer was well tolerated. No significant adverse events were reported. The administered activity was approximately 370 MBq for each patient (activity dispensed by the Radiopharmacy before injection: median 388 MBq).

Main clinical characteristics were mean age 63 years ± 5.8 (range 51–72), 89% high and 11% very high risk (D’Amico risk classification) [1] and mean PSA 9.1 ± 6.1 ng/mL (range 4.2–25), whereas main pathological characteristics were pT3a in 53% of patients, positive surgical margins in 47%, extracapsular/seminal vesicle/perineural involvement respectively in 74%, 21% and 84%; pGS 4 + 3 in 42% and 4 + 5 in 37%. Overall patients’ characteristics are presented in detail in Table 1.

For both conventional and investigational tracers, PET uptake was documented in the prostate gland in all patients; asymmetrical uptake in the prostate that exceeded the surrounding background activity was visualized in all patients. In particular, the location of the “blinded” prostate SUVmax matched with the lobe of the index lesion (highest pGS) in 17/19 cases (89%). Only in 2/19 (11%) patients the area of maximum uptake was found in a prostatic lobe not identified as malignant by the pathologist on the reference template. Mean MTV with [^18^F]Fluciclovine was 20.75 ± 9.76 cm^3^, while with [^11^C]Choline, 17.60 ± 8.09 cm^3^.

### 3.2. Lesion-Based Analyses (Unblinded to Histopathology, According to the Uro-Pathologist Prostate Template)

According to the pathological result of RP (graphically reported on the uro-pathologist reference prostate template), overall, 45 malignant and 31 benign lesions were found; furthermore, a single area of healthy prostate tissue was selected for each patient (*n =* 19). A total number of 95 areas were therefore included in the final lesion-based analyses.

Among the 45 malignant lesions, the pISUP was distributed as following: 1,2,3,4 and 5 in 12, 12, 11, 3 and 10 lesions, respectively. In all cases conventional acinar prostate adenocarcinoma was found, except for one single neuroendocrine tumor. Malignant lesions (*n =* 45) had a median SUV max of 5 (mean 5.55 ± 2.68, range 2.3–14.6) with [^11^C]Choline, whereas of 5.10 (mean 5.47 ± 2.33; range 0.6–3.9) with [^18^F]Fluciclovine.

A statistically significant correlation between semi-quantitative PET parameters and pISUP of the 45 malignant lesions was found when using SUVmax (*p* < 0.001), TBR-L3 (*p* = 0.01) and TBR-LIVER (*p* < 0.001) for [^18^F]Fluciclovine and only when using TBR-LIVER for [^11^C]Choline (*p* < 0.001) (Table 2). The higher were the values of these indicators, the higher the pISUP was at final pathology. To note that TBR-L3 was close to be statistically significant (*p* = 0.06) also with [^11^C]Choline. On the contrary this correlation was not statistically significant when using [^11^C]Choline SUVmax or when aorta was considered as reference background for TBR (TBR-AORTA) with both radiotracers.

Median (IQR) values of PET parameters (SUV max, TBR-Aorta, TBR-L3, TBR-Liver) are listed in Table 3.

When PET parameters of the three different types of areas (healthy/benign/malignant) were analyzed contemporary applying Kruskal–Wallis test, a statistically significant difference was pointed out with both tracers (*p* < 0.001, Table 3), with a clearer, though minimal, separation between malignant and benign lesion group with [^18^F]Fluciclovine. Furthermore, they all resulted significantly higher (*p* < 0.001, Table 3) in malignant lesions than non-malignant ones (considering benign lesions grouped with healthy tissue).

SUV max was not significantly higher in malignant than benign lesions (*p* = 0.12 and *p* = 0.10) but was significantly higher in malignant than healthy tissue (*p* < 0.001) for [^18^F]Fluciclovine and [^11^C]Choline, respectively). Regarding TBR parameters, a statistically significant difference between malignant and benign lesions was highlighted for [^18^F]Fluciclovine when the reference background was measured at the level of abdominal aorta (TBR-AORTA, *p* = 0.016) and L3 (TBR-L3, *p* = 0.009), but not when it was calculated at the level of healthy prostate tissue (*p* = 0.26) and liver (TBR-LIVER, *p* = 0.183). On the contrary, [^11^C]Choline TBR of malignant lesions were significantly higher than benign ones only when this was measured at the level of the abdominal aorta (*p* = 0.0138).

Among the 31 benign lesions, the following subtypes were identified: 6 PIN, 16 inflammation, 6 PAH, 2 inflammation + PAH and 1 prostate infarction zone. The average, SD, median and range for PET uptake values, with [^18^F]Fluciclovine and [^11^C]Choline, for each different benign histo-type are presented in Supplementary Materials [Table S1: [^18^F]Fluciclovine and [^11^C]Choline PET uptake values (mean ± standard deviation; median; range) for each benign histo-type (*n =* 31)].

**Figure 1:**
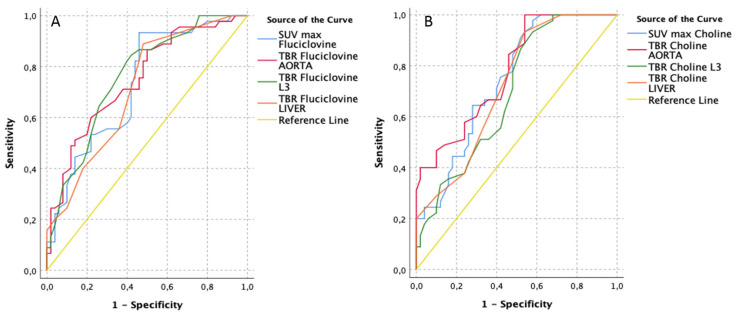
Receiver operating characteristics (ROC) curves of [^18^F]Fluciclovine (**A**) vs. [^11^C]Choline PET/CT (**B**) performance for malignant lesion detection, using semi-quantitative parameters (lesion based, *n =* 45/95).

The diagnostic performance, in terms of AUC to discriminate between a malignant lesion (*n =* 45/95) from a non-malignant one, resulted moderate and similar for each semi-quantitative parameter of both tracers (AUCs between 0.70 and 0.78, Figure 1 and Table 4). Although suboptimal, a slight superiority was found for [^11^C]Choline TBR-AORTA (AUC 0.78), reaching a 100% sensitivity and 46% specificity when using 2 as best cut-off value (Youden index applied to ROC curves) and for [^18^F]Fluciclovine TBR-L3 (AUC 0.76), leading to 84% sensitivity and 58% specificity with a threshold of 1.35.

**Figure 2:**
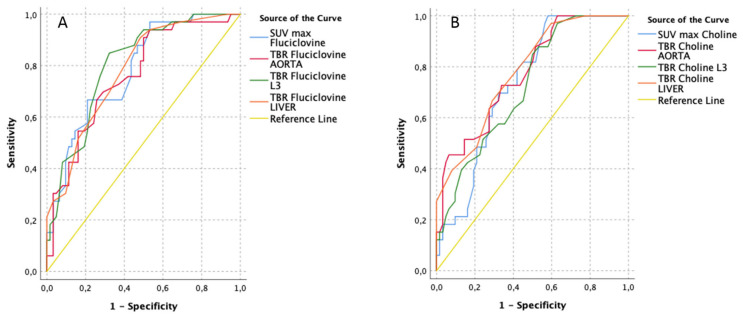
Receiver operating characteristics (ROC) curves of [^18^F]Fluciclovine (**A**) vs. [^11^C]Choline PET/CT (**B**) performance for clinical significant lesion (pISUP 2–5) detection, using semi-quantitative parameters (lesion-based, *n =* 33/95).

The ability of detection of a clinically significant (ISUP 2–5) lesion (*n =* 33/95) was also assessed (Figure 2 and Table 5), showing a slightly better performance of the investigational tracer (AUCs ranging from 0.76 of TBR-AORTA to 0.80 of TBR-L3) in comparison with the standard tracer (AUCs ranging from 0.72 of TBR-L3 to 0.78 of TBR-LIVER). In particular, a [^18^F]Fluciclovine TBR-L3 ≥ 1.5 would depict a clinical significant lesion with a sensitivity and specificity of 85% and 68% respectively.

**Figure 3:**
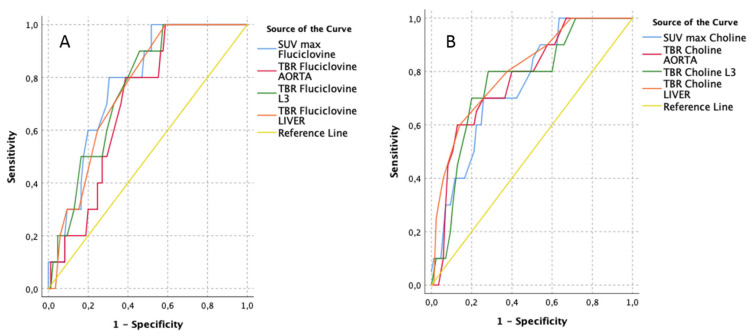
Receiver operating characteristics (ROC) curves of [^18^F]Fluciclovine (**A**) vs. [^11^C]Choline PET/CT (**B**) performance for high-grade malignant lesions (pISUP 4–5) detection, using semi-quantitative parameters (lesion-based, *n =* 10/95).

On the contrary, when the diagnostic performance for high-grade (ISUP 4–5) lesion (*n =* 10/95) detection was evaluated (Figure 3 and Table 6), slightly better results were found for [^11^C]Choline (AUCs ranging from 0.75 to 0.81), in particular using either TBR-LIVER (AUC 0.81; best cut-off 0.75 showing 60% sensitivity and 86% specificity) or TBR-AORTA (AUC 0.78; best cut-off 4.85 showing 60% sensitivity and 87% specificity). Concerning [^18^F]Fluciclovine parameters (AUCs ranging from 0.70 to 0.77), this was the only case in which SUVmax (AUC 0.77) outperformed TBRs; a cut-off value of 4 would be able to identify a ISUP 4–5 lesion in all cases (sensitivity 100%), although with low specificity (52%).

**Figure 4:**
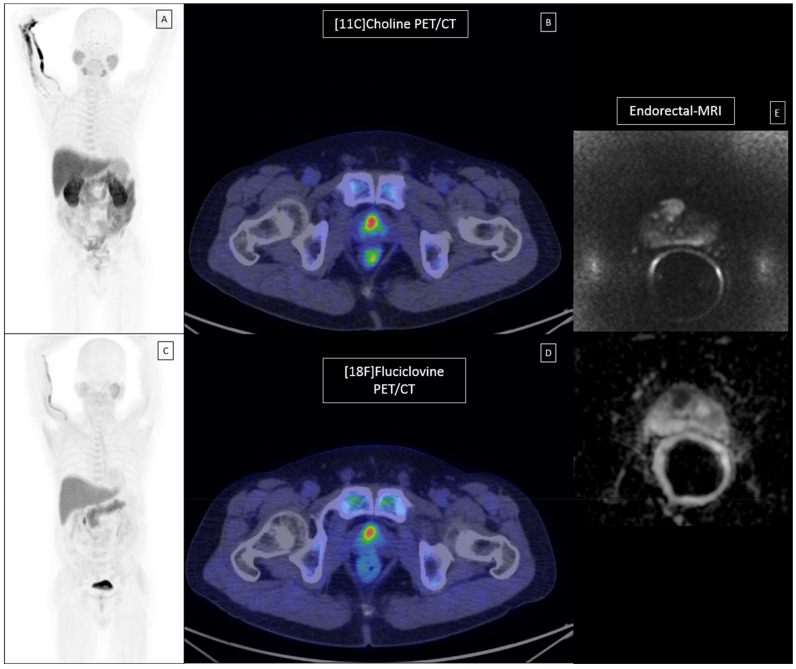
Concordant [^11^C]Choline, [^18^F]Fluciclovine PET/CT and mpMRI in a ISUP 5 PCa lesion. (**A**) A patient affected by high-risk primary Pca underwent conventional [^11^C]Choline PET/CT (A-mip; B- axial fused PET/CT), additional investigational [^18^F]Fluciclovine PET/CT (C-mip; D-axial fused PET/CT) and conventional mpMRI (E- axial DWI and ADC) before RP. (**B**) Staging imaging resulted positive in middle-apex right prostate lobe in all cases, corresponding with the most aggressive high-grade lesion (pGS 4 + 5; pISUP5). (**C**) The lesion uptake was clearly above the bone marrow background (measured at L3 vertebral body) with both tracers. (**D**) A further millimetric malignant focus of pGS 3 + 3 was identified at histopathological analyses but it was not detected at imaging

An example of concordant [^11^C]Choline, [^18^F]Fluciclovine PET/CT and mpMRI focal positivity, corresponding with a high-grade (ISUP 5) PCa lesion at final pathology, is presented in Figure 4. As expected, a millimetric and low-grade malignant focus (pGS 3 + 3) was not detected by imaging.

At univariate analyses (lesion-based, Table 7), each PET semi-quantitative parameter resulted a significant predictor of malignancy with both tracers (*p* < 0.001 in all cases; except for [^11^C]Choline TBR-L3 with *p* = 0.002). On the contrary clinical parameters (iPSA, cT, cISUP, PIRADS v.2) were not significant predictive factors. For [^11^C]Choline, TBR-AORTA (OR: 6.12) resulted an independent predictor of malignancy at multivariate logistic regression analysis (*p* = 0.01). On the other hand, TBR-L3 was very close to the statistical significance with both tracers ([^18^F]Fluciclovine *p* = 0.06; [^11^C]Choline *p* = 0.05).

## 4. Discussion

### 4.1. Scientific Literature

In addition to the initial, preliminary studies reported in the “Introduction” section [14,18,28], further works have been published, during our study enrollment and afterwards, about [^18^F]Fluciclovine diagnostic performance in primary PCa. Elschot et al. [34] extracted Voxel and VOI features from 40 tumors (26 high-grade), 36 BPH, 6 prostatitis and 37 healthy-tissue, in 28 pts, observing relatively small absolute differences in SUVs between tumors and benign lesions, indicating that PET images alone may not be sufficient for evaluation of primary PCa in clinical practice; nevertheless, the discriminative power of PET was strong enough to improve MRI-based classification by approximately 5% when combined. The FLUCIPRO Trial was conducted in 26 intermediate to high-risk PCa pts: quantitative [^18^F]Fluciclovine imaging failed to outperform MRI in lesion detection; however, it significantly correlated with GS suggesting a potential role in assisting targeted biopsies in the setting of hybrid imaging with MR [35]. In a recent systematic review and meta-analysis by Kim and Lee, across 13 studies (563 pts), the pooled sensitivity for [^18^F]Fluciclovine PET/CT for diagnosis of primary PCa was 0.87 (95 CI: 0.77–0.93) and the pooled specificity 0.84 (95% CI: 0.68–0.93) [36].

Considering the standard radiolabelled-Choline, previous studies already reported that the difference between [^11^C]Choline SUVs of PCa and BPH were not statistically significant but with a tendency towards higher values in malignant lesions [11]. Although equivocal results in terms of specificity, the sensitivity for prostatic tumor foci can be high enough to complement MRI findings in the final diagnosis. In a study by Hernández-Argüello M. et al., the Index lesion was defined as the largest tumor measured on histopathology (in our study is the one with either the highest pGS or higher pISUP); the sensitivity, specificity, negative and positive predictive value (NPV and PPV) for tumor detection were 100%, 70%, 83%, 100%, for PET and 46%, 100%, 100%, 54% for MRI, respectively; both Index-PET and Index-MRI were complementary and identified 95% of the Index-lesions when quantitative criteria (SUVmax and apparent diffucion coefficient-ADC) were used [37].

Simultaneous [^18^F]Choline PET/MRI showed a better diagnostic value for localized PCa detection than each individual modality [38] and no correlation between SUVs and ADC hypothesized that uptake features characterize different parts of tumor biology respect to MRI ones [39].

Combined dynamic [^18^F]Choline PET/MR imaging showed, in 12 intermediate to high-risk PCa patients, good individual correlations between SUVmax and PIRADS score with several clinical-pathologic characteristics; on the contrary no clear clinical relevance was found for dynamic acquisitions [40]. In contrast, Schaefferkoetter et al. demonstrated that dynamic parameters (i.e., SUV,K1,Ki and Patlak slope), although poor differentiators between low-grade tumor and healthy prostate tissue, represent strong indicators of aggressive disease [41].

Another metabolic PET tracer, [^11^C]Acetate, is of limited value due to low diagnostic performance (pooled sensitivity 75.1% [69.8–79.8] and specificity 75.8% [72.4–78.9] [42].

Among high-grade PCa, potentially exhibiting increased glycolytic rate, PET/CT with [^18^F] FDG could improve pre-treatment prognostic stratification by predicting pathological grade and survival probability; however, to date, its application in clinical practice remains restricted to a very few circumstances [43].

An alternative imaging in PCa, with outstanding development in recent years and high accuracy, is represented by PET/CT with radiolabelled-PSMA (see Section 4.4.).

### 4.2. Considerations on the Present Study

To the best of our knowledge, despite the small sample size, this is the first study investigating [^18^F]Fluciclovine performance in the characterization of intraprostatic lesions in high-risk primary PCa patients with surgical pathologic confirmation in which comparison with standard [^11^C]Choline is available. In the present study, 19 high-risk PCa patients underwent [^18^F]Fluciclovine PET/CT in addition to the standard staging work-up, which routinely included [^11^C]Choline PET/CT (according to the risk-class and the large availability in our high-volume diagnostic PET Center).

The novelty of our study is that the attention was focused not only on the overall diagnostic PET/CT performance, but especially on the role of multiple semi-quantitative metabolic parameters of the relatively new [^18^F]Fluciclovine and the largely diffuse conventional choline tracers, whose interpretation and positivity criteria in primary PCa setting are not well defined/standardized yet.

The already known overlap of uptake values between malignant and benign lesions was confirmed also by our results (Table 3); therefore, none of the tracers was able to accurately discriminate between malignant and benign lesions. However, we confirmed a significant uptake difference between malignant and healthy tissue and demonstrated a significant difference when the three types of areas (healthy/benign/malignant) were analyzed contemporary. Furthermore, when benign lesions were grouped with normal prostate zones as a more general “non-malignant” category, all semi-quantitative parameters resulted significantly higher in the malignant group. This study also proved the direct correlation between [^18^F]Fluciclovine uptake values (in terms of SUVmax and TBRs, except when using blood pool as reference background) and pathological grading (Table 2), as already reported by previous studies by Schuster et al. [14,18,29] and Jambor and colleagues [35], although in our case expressed in terms of pISUP instead of pGS. Thus, as speculated by previous publications, we confirm the hypothesis of a potential role of PET with metabolic tracers, complementary to transrectal ultrasound (TRUS)/MRI imaging, in leading prostatic biopsy to detect the most aggressive focus (higher pGS/pISUP).

Overall, in our setting, the performance of PET with either [^18^F]Fluciclovine or [^11^C]Choline in detecting either malignant/ISUP2–5/ISUP4–5 PCa lesions is moderate (AUCs resulted ≥0.70) but still inadequate (AUCs never reached values beyond 0.81) as a standalone staging procedure. Furthermore, performances of the investigational [^18^F]Fluciclovine were very similar to the conventional [^11^C]Choline, with no definite superiority of any particular parameter nor in any particular setting (malignant vs. clinical significant lesion vs. high-grade lesion detection) over the other ones. These findings hamper clinical decision based on [^18^F]Fluciclovine PET images only and seriously threatens its applicability as a standalone modality in this setting. Unquestionable favorable characteristics and practical/technical advantages of the new radiopharmaceutical should be taken into account; however, a clear suggestion of [^18^F]Fluciclovine as a substitute tracer based on diagnostic performance would not be justified, thus, remaining more an alternative option to standard choline indications in centers without cyclotron onsite.

Important implications derive from multivariate logistic regression analyses revealing that TBR-L3 was very close to the statistical significance with both tracers. For [^11^C]Choline, TBR-AORTA represented an independent predictor of malignant lesion. Slight differences between the two tracers might be related to variability in para-physiological biodistribution at the level of background structures selected for TBRs calculations. For instance, faint, homogeneous, diffuse uptake in the bone marrow, due to unspecific activation, is frequently seen with [^18^F]Fluciclovine but it is a more common finding especially with choline, explicating a minor inferiority of the corresponding TBR-L3 AUCs performances of the standard tracer compared to the investigational one. Secondly, considering blood pool, the recommended uptake time for [^18^F]Fluciclovine scanning was 3–5 min with the specific goal of 4 min, which was respected, in line with the investigational prospective protocol. On the other hand, for the standard tracer, a comparable but not strictly as fast uptake time is indicated, encountering also the possibility of a few minutes of delay in PET scanning start in the daily diagnostic routine; although minimal, this time delay might have reflected into slightly increased [^11^C]Choline extraction from the blood pool and lower uptake values in aorta, that would explain the overall better diagnostic application of TBR-AORTA parameter in [^11^C]Choline scan respect to [^18^F]Fluciclovine. Another factor potentially influencing efficacy dissimilarities is a possibly different tracer-dependent accumulation between acute and chronic inflammation (this further sub-classification of phlogistic areas-*n =* 18-lacked at our final pathology examinations), already documented in pre-clinical studies with [^18^F]Fluciclovine showing higher uptake in chronic vs. acute inflammed lymph-nodes in rats [44], an issue apparently less explored with choline.

At the time of our study design and start, there were no clear recommendations for [^18^F]Fluciclovine PET imaging interpretation. More recently, [^18^F]Fluciclovine uptake became suspicious for malignancy in the prostate district when diffuse, focal, or multi-focal uptake is greater than the bone marrow. However, in previous studies, bone marrow frequently appeared moderate and heterogeneous with the aminoacid compound (in keeping with location of red marrow), occasionally representing a complex reference background [29,45]. According to guidelines [46], although uptake between blood pool and bone marrow does not meet definite criteria for malignancy, it may still be reported as suspicious, especially when the intraprostatic focus of [^18^F]Fluciclovine is small (<1 cm; partial volume effect); MRI correlation is especially helpful in this situation. To note that these criteria derive from experience in the setting of recurrent PCa and therefore are not directly applicable to the setting of staging primary PCa. Refinement of these criteria is warranted in order to improve the performance, as well as reader training implementation when investigating a new tracer [47].

Liver, an important background structure when it comes to [^18^F] FDG (i.e., to define Deauville Score criteria for aggressive lymphomas and multiple myelomas), was also included in our analyses despite its high, physiological uptake with both [^11^C]Choline and [^18^F]Fluciclovine: although TBR-Liver performance resulted significant, the best cut-offs were always <1, meaning that the malignant lesion uptake was significantly lower than the liver, unlikely representing an appropriate reference background.

Regarding choline, the main published studies were restricted to the exclusive evaluation of apparent tumor SUV (mean or maximum) and a few of them reported no correlation between [^11^C]Choline SUVmax and the histological grade, GS, volume of the prostate or PSA [25]. Finally, a ratio of lesion SUVmax with SUVmax of pelvic muscle was proposed for the tracer [^11^C]Choline; although higher in GS ≥ 4 + 3 and significantly associated with clinical tumour stage and GS in a small population of 26 pts histologically validated, this TBR criteria was not routinely included in clinical practice [48].

Considering our ROC analyses (Table 4, Table 5 and Table 6), we would not recommend/support a definite threshold for the detection of malignant prostatic lesion. Image interpretation should be based mainly on qualitative characteristics; however, to implement an objective delineation of the target malignant lesion to guide focal therapy approaches or biopsies, our results seem to point towards complementary algorithms based on TBRs cut-offs, which may be helpful. The already known positivity criteria of lesion uptake greater than the bone marrow seems reliable because we found, as best cut-offs, TBR-L3 values between 1.35 and 1.55 and TBR-AORTA values between 2.7 and 3.75, meaning that a malignant lesion usually shows uptake significantly greater (double/triple) than blood pool and slightly higher than bone marrow (and still lower than liver).

### 4.3. Limitations of the Present Study

The present study is affected by several limitations: first of all, the small sample size (*n =* 19 pts); however, statistical analyses were performed lesion-based (*n =* 95) to improve the investigation of [^18^F]Fluciclovine in detecting primary malignant prostatic lesions; secondly, the nature of the study cohort itself, in which only high-risk patients were examined. Accurate local T staging is of major importance for this group of patients; on the other hand, very-high-risk patients with conventional imaging positive for extra-pelvic and distant metastases were not eligible for this study due to surgical exclusion (this population was chosen in order to get tissue confirmation on virtually all patients). Therefore, the transferability of our results and the utility in different risk disease have yet to be assessed, not conveying the impact in global primary PCa setting.

Ideally, our sample size could have been larger, but this possibility was abandoned due to following reasons: the lack of pathological validation either in high-risk M1 patients, or in patients addressed to radiotherapy; the lack of an additional more accurate histo-pathological analysis of prostatectomy for lesion-based analysis, specifically aimed at mapping the precise location, nature and type of each malignant and benign intraprostatic lesion, which was dedicated only to patients who underwent all standard pre-operative staging investigations (including mpMRI) at our center.

The diagnostic performance of the competitor mpMRI for local staging was not part of our objectives and was not taken into consideration in our analyses. Measuring the exact tumor extension, not only location and assessing mpMRI features in each selected intra-prostatic area in comparison with PET and histopathology, could be part of further analyses.

Searching for clinical-diagnostic features at initial staging predicting for PCa recurrence was not in our purposes, therefore, a follow-up surveillance after surgical pathological examination was not included.

The optimal timing of [^18^F]Fluciclovine PET to best differentiate between prostate tumors and benign tissue, as well as between high and low/intermediate-grade, was found in the late-window, at 33–38 min [49] or 28 min [18] p.i. In contrast, it is well known that immediate [^18^F]Fluciclovine uptake and relatively fast wash-out is seen in metastatic lymph nodes. The patients included in our present study were selected among those ones enrolled in a prospective trial aimed at nodal staging. We can therefore, speculate that our early window (PET 3–5 min p.i.) was set as optimal, for lymph node metastases detection but potentially suboptimal for primary tumor assessment, especially in terms of specificity. However, the peak of [^18^F]Fluciclovine kinetic activity in PCa was proven exactly at 4.5 min in previous studies [17], therefore, the assessment at this time frame should have optimized sensitivity and is in line with current guidelines recommendation [46]. In addition, a dual time acquisition protocol, combining early and late images, would have led to a time-consuming procedure and to more patient’s discomfort.

The failure in detecting small, millimetric intraprostatic cancer deposits due to low metabolic uptake, are expected to be related mainly to intrinsic limitations of PET method (the partial volume averaging and the limited spatial resolution of approximately 5 mm), rather than to the tracer characteristics.

Lack of accurate co-registration of histo-pathology is a limiting step of our process, in particular due to the poor correlation between slice thickness and to orientation. However, in our case, validation was not driven by a subjective, visual approach because we tried to overcome this limitation through a sector-based prostate reference template, facilitating the direct anatomical mapping of pathology onto imaging [50]. Additionally, to the clinical workup, the pathologist was asked to manually delineate intra-prostatic lesions on the prostate template according to the histological slices; then, the nuclear medicine physician followed the contours defined by the pathologist and projected onto the corresponding PET slices in order to measure the semi-quantitative parameters in the exact corresponding location. This approach was similar to that used in previous works [25,41,51,52]. A few new pathology/imaging co-registration techniques have been implemented in the last years [53], i.e., a sophisticated co-registration allowing voxel-wise analysis between PET scans and histopathology [54]. To note that several uncertainties remain also in co-registration, mainly related to differences in imaging and histology resolution and to shrinkage factors between in-vivo and ex-vivo and that, in general, there is no ground truth for registration accuracy.

Surprisingly, there were no clinical parameters (iPSA, cT, cISUP, PIRADS), at univariate analysis, significantly predictive for malignant lesions. However, overall patients included harboured high-risk PCa (as requested by inclusion criteria). In contrast, each patient has multiple lesion-based PET semi-quantitative variables due to multiple measurable areas of interest (of different nature and risk) in each patient prostate gland. Thus, when clinical parameters are analyzed with a lesion based-method, there is a potential bias and the reliability of clinical data results is affected.

Moreover, further multivariate regression analysis to predict clinically significant ISUP 2–5 lesion or high grade (ISUP-4–5) lesions were not employed, due to the limited cohort with risk to generate over-fitted models.

### 4.4. Recent Research and Future Developments in Multimodality Imaging of Primary PCa

After initiation of this study, PSMA, has emerged as a tracer of choice for radionuclide imaging of PCa [55]. It can be labeled both with [68Ga], which does not require a cyclotron on-site and [^18^F], characterized by a longer half-life. In addition, being not metabolic but related to the extent of expression of PSMA, it seems to perform better not only in BCR but also in primary PCa setting [56]. The tumor-to-non-tumor ratio in the prostate gland improves over time, supporting a role of delayed imaging for optimal visualization of PCa [57]. Small lesions under the spatial resolution of PET may still be missed. Similar specificity scores to mpMRI (approximately 85%) and slightly higher sensitivity (76%) were reported. [68Ga]PSMA already plays a role in the setting of targeted biopsy after previous negative biopsy in pts with high suspicion of PCa [55]. Preferably, it should be combined with mpMRI for a multimodality approach, allowing: (a) image-guided biopsy [58]; (b) increased diagnostic confidence [56,59,60]; (c) improved gross tumor volume (GTV) delineation. Additionally, [68Ga]-PSMA PET is also beneficial for N and M staging [61]. The SUVmax of the primary tumor has a relation with GS, metastatic extent of disease and PSA levels, defining the prognosis [62]. SUVmax values correlated significantly with the Grade Group of the primary tumor of 141 pts in a retrospective study [63]. Wang et al. established cutoff values of ADC, SUVmax and SUVmax/ADC at 1.02 × 10 mm/s, 11.72 and 12.35, respectively, to differentiate PCa from benign lesions [64]. However, whether 68Ga-PSMA is superior to [^18^F]Fluciclovine in diagnosis of early PCa requires further confirmatory studies, hopefully in the setting of head-to-head comparison in patients receiving both tracers before prostatectomy.

The development of combined PET/MRI, merging morphological detail and multi-parametric functional data with molecular PET information, allows to a “one-step” imaging for T staging and targeted-biopsy approach, reduced radiation exposure, shorter (cumulative) scan times and intrinsic alignment of PET and MR images; however, the availability is limited by unaffordable high costs [65]. A recent meta-analysis on PET/MRI by Evangelista et al. showed that initial disease staging was the main indication in 24 studies and radiolabeled PSMA was the tracer most frequently used: in primary tumors, the pooled sensitivity for the patient-based analysis was 94.9% [66].

Biological characterization of tumor aggressiveness could be further explored through Diffusion Weighted Imaging (DWI) or Dynamic contrast enhanced (DCE)-MRI sequences, radiomics and deep learning classifiers [67,68,69], quantitative approaches and computer-aided diagnostic systems.

A precise and earlier risk stratification in the natural history of PCa could be beneficial for therapeutic decision making; thus, incorporating PET imaging at an earlier phase of the clinical-diagnostic staging workup of PCa might improve patient management.

## 5. Conclusions

In our setting of primary high-risk PCa, the location of the highest SUVmax matched with the lobe of the index lesion (highest pGS) in 17/19 cases (89%). On a lesion-basis, there was direct correlation between [^18^F]Fluciclovine uptake values and pathological grading. Overall, the performance of PET semiquantitative parameters with either [^18^F]Fluciclovine or [^11^C]Choline, in detecting either malignant/ISUP2–5/ISUP4–5 PCa lesions, was moderate and similar (AUCs resulted ≥0.70), but still inadequate (AUCs never reached values beyond 0.81) as a standalone staging procedure. TBRs (especially with threshold significantly higher than aorta and slightly higher than bone marrow), may be complementary to implement malignancy targeting.

## Figures and Tables

**Table 1:** Patients’ characteristics (*n =* 19).

Age (years)	Mean	63.4
	SD	5.8
	Median	64
	Range	51–72
iPSA (ng/mL)	Mean	9.1
	SD	6.1
	Median	6.6
	Range	4.2–25
DRE	Negative	6/19 (32%)
	Positive	2/19 (10%)
	n/a	11/19 (58%)
TRUS	Negative	4/19 (21%)
	Positive	12/19 (63%)
	n/a	3/19 (16%)
PI-RADS v.2	3	3/19 (16%)
	4	2/19 (10%)
	5	8/19 (26%)
	n/a	6/19 (32%)
Positive cores	Mean	54%
	SD	18%
	Median	0.5%
	Range	25–100%
cGS	3 + 4	2/19 (10%)
	4 + 3	1/19 (1%)
	4 + 4	10/19 (53%)
	4 + 5	6/19 (32%)
cT	1	2/19 (10%)
	2	17/19 (89%)
pT	2c	5/19 (26%)
	3a	10/19 (53%)
	3b	4/19 (21%)
pGS	4 + 3	5/19 (26%)
	4 + 4	5/19 (26%)
	3 + 5	1/19 (5%)
	4 + 5	7/19 (37%)
	5 + 4	1/19 (1%)
R1	No	10/19 (53%)
	Yes	9/19 (47%)
ECE	Yes	14/19 (74%)
	No	5/19 (26%)
SVI	Yes	4/19 (21%)
	No	15/19 (79%)
Perineural Invasion	Yes	16/19 (84%)
	No	3/19 (16%)
pN	0	14/19 (74%)
	1	5/19 (26%)

Legend: initial Prostate Specific Antigen (iPSA); digital rectal examination (DRE); transrectral ultrasound (TRUS); prostate imaging–reporting and data system (PI-RADS); clinical Gleason Score (cGS); clinical T (cT); pathological Gleason score (pGS); R1= positive surgical margins at histopatholy; extracapsular extension (ECE); seminal vesicle invasion (SVI); pathological nodal staging (pN).

**Table 2:** Linear correlation test between [^18^F]Fluciclovine and [^11^C]Choline and pISUP of malignant lesions (*n =* 45).

Spearman Correlations (*n =* 45)		SUV Max Choline	SUV Max Fluciclovine	TBR Choline L3	TBR Fluciclovine L3	TBR Choline LIVER	TBR Fluciclovine LIVER	TBR Choline AORTA	TBR Fluciclovine AORTA
pISUP	Coeff	0.22	0.46	0.28	0.37	0.45	0.47	0.22	0.26
	*p*	0.15	**<0.001**	*0.06*	0.01	**<0.001**	**<0.001**	0.15	0.08

Legend: Target to Background Ratio (TBR); L3 (bone marrow background measured at L3 vertebral body); pathological International Society of Urological Pathology (pISUP). *p* < 0.05 was considered the threshold for statistical significance and reported in bold. *p* = 0.06 is reported in italics being close to the statistical significance.

**Table 3:** Semi-quantitative PET parameters in malignant vs. non-malignant lesion and malignant lesion vs. benign lesion vs. normal prostatic tissue, considering final pathologic examination as reference standard (lesion-based analysis; *n =* 95).

PET Parameters	Malignant (*n =* 45)	Non-Malignant (*n =* 50)	*p* Value	Malignant (*n =* 45)	Benign (*n =* 31)	Normal Prostatic Tissue (*n =* 19)	*p* Value
SUV max Choline Median (IQR)	5 (3.6–6.2)	3.2 (1.9–5.2)	**0.001**	5 (3.6–6.2)	4.5 (3.1–5.8)	2.1 (1.7–2.6)	**<0.001**
SUV max Fluciclovine Median (IQR)	5.1 (3.9–6.3)	2.8 (2.4–4.8)	**<0.001**	5.1 (3.9–6.3)	4.7 (2.8–5.2)	2.3 (2–2.8)	**<0.001**
TBR aorta Choline Median (IQR)	3.7 (2.6–5.4)	2.4 (1.5–3.3)	**<0.001**	3.7 (2.6–5.4)	3.1 (2.4–3.7)	1.6 (1.3–2.3)	**<0.001**
TBR aorta Fluciclovine Median (IQR)	4 (3.1–5.4)	2.5 (1.5–3.7)	**<0.001**	4 (3.1–5.4)	3.6 (2.2–3.9)	1.6 (1.3–2.3)	**<0.001**
TBR L3 Choline Median (IQR)	1.7 (1.2–2.2)	1.1 (0.6–1.8)	**<0.001**	1.7 (1.2–2.2)	1.7 (0.9–2)	0.8 (0.6–0.9)	**<0.001**
TBR L3 Fluciclovine Median (IQR)	1.8 (1.5–2.2)	1.1 (0.6–1.7)	**<0.001**	1.8 (1.5–2.2)	1.5 (1.1–2)	0.8 (0.5–0.9)	**<0.001**
TBR Liver Choline Median (IQR)	0.6 (0.5–0.8)	0.4 (0.2–0.6)	**<0.001**	0.6 (0.5–0.8)	0.6 (0.4–0.7)	0.3 (0.2–0.3)	**<0.001**
TBR Liver Fluciclovine Median (IQR)	0.7 (0.6–0.9)	0.4 (0.3–0.7)	**<0.001**	0.7 (0.6–0.9)	0.7 (0.4–0.8)	0.3 (0.3–0.3)	**<0.001**

Legend: Target to Background Ratio (TBR); L3 (bone marrow background measured at L3 vertebral body); Interquartile range (IQR). *p* < 0.05 was considered the threshold for statistical significance and reported in bold.

**Table 4:** Comparison of [^18^F]Fluciclovine vs. [^11^C]Choline PET/CT performance for malignant lesion detection, using semi-quantitative parameters (lesion based, *n =* 45/95). Areas under the ROC curves (AUCs) are listed (in bold) in descending order. Best cut-off values for each parameter and corresponding sensitivity (sens) and specificity (spec) are also presented.

[^18^F]Fluciclovine PET/CT Parameters	AUC	Std. Error	Asymptotic Sig.	Asymptotic 95% Confidence Interval		Best Cut-Off (Youden-Index)			[^11^C]Choline PET/CT Parameters	AUC	Std. Error	Asymptotic Sig.	Asymptotic 95% Confidence Interval		Best Cut-Off (Youden-Index)		
				Lower Bound	Upper Bound	Value	Sens	Spec					Lower Bound	Upper Bound	Value	Sens	Spec
TBR Fluciclovine L3	**0.76**	0.05	0.00	0.66	0.86	1.35	0.84	0.58	TBR Choline AORTA	**0.78**	0.05	0.00	0.69	0.87	2.00	1.00	0.46
TBR Fluciclovine AORTA	**0.75**	0.05	0.00	0.65	0.85	3.75	0.60	0.78	SUV max Choline	**0.74**	0.05	0.00	0.65	0.84	2.75	0.93	0.46
SUV max Fluciclovine	**0.73**	0.05	0.00	0.63	0.83	3.05	0.93	0.54	TBR Choline LIVER	**0.72**	0.05	0.00	0.62	0.82	0.35	0.93	0.46
TBR Fluciclovine LIVER	**0.71**	0.05	0.00	0.61	0.82	0.45	0.89	0.52	TBR Choline L3	**0.70**	0.05	0.00	0.59	0.80	0.85	0.93	0.42

**Table 5:** Comparison of [^18^F]Fluciclovine vs. [^11^C]Choline PET/CT performance for clinical significant lesion (pISUP 2–5), using semi-quantitative parameters (lesion based, *n =* 33/95). Areas under the ROC curves (AUCs) are listed (in bold) in descending order. Best cut-off values for each parameter and corresponding sensitivity (sens) and specificity (spec) are also presented.

[^18^F]Fluciclovine PET/CT Parameters	AUC	Std. Error	Asymptotic Sig.	Asymptotic 95% Confidence Interval		Best Cut-Off (Youden-Index)			[^11^C]Choline PET/CT Parameters	AUC	Std. Error	Asymptotic Sig.	Asymptotic 95% Confidence Interval		Best Cut-Off (Youden-Index)		
				Lower Bound	Upper Bound	Value	Sens	Spec					Lower Bound	Upper Bound	Value	Sens	Spec
TBR Fluciclovine L3	**0.80**	0.04	0.00	0.72	0.89	1.55	0.85	0.68	TBR Choline LIVER	**0.78**	0.05	0.00	0.69	0.87	0.55	0.67	0.71
SUV max Fluciclovine	**0.78**	0.05	0.00	0.69	0.88	3.05	0.97	0.47	TBR Choline AORTA	**0.77**	0.05	0.00	0.68	0.87	4.60	0.45	0.94
TBR Fluciclovine LIVER	**0.78**	0.05	0.00	0.69	0.87	0.55	0.91	0.52	SUV max Choline	**0.73**	0.05	0.00	0.63	0.83	2.75	1.00	0.42
TBR Fluciclovine AORTA	**0.76**	0.05	0.00	0.66	0.86	2.70	0.91	0.50	TBR Choline L3	**0.72**	0.05	0.00	0.62	0.83	1.15	0.85	0.50

**Table 6:** Comparison of [^18^F]Fluciclovine vs. [^11^C]Choline PET/CT performance for high-grade malignant lesions (pISUP 4–5) detection, using semi-quantitative parameters (lesion based, *n =* 10/95). Areas under the ROC curves (AUCs) are listed (in bold) in descending order. Best cut-off values for each parameter and corresponding sensitivity (sens) and specificity (spec) are also presented.

[^18^F]Fluciclovine PET/CT Parameters	AUC	Std. Error	Asymptotic Sig.	Asymptotic 95% Confidence Interval		Best Cut-Off (Youden-Index)			[^11^C]Choline PET/CT Parameters	AUC	Std. Error	Asymptotic Sig.	Asymptotic 95% Confidence Interval		Best Cut-Off (Youden-Index)		
				Lower Bound	Upper Bound	Value	Sens	Spec					Lower Bound	Upper Bound	Value	Sens	Spec
SUV max Fluciclovine	**0.77**	0.06	0.00	0.66	0.89	4.00	1.00	0.52	TBR Choline LIVER	**0.81**	0.07	0.00	0.67	0.94	0.75	0.60	0.86
TBR Fluciclovine LIVER	**0.76**	0.06	0.00	0.64	0.88	0.55	1.00	0.59	TBR Choline AORTA	**0.78**	0.07	0.00	0.63	0.92	4.85	0.60	0.87
TBR Fluciclovine L3	**0.76**	0.06	0.00	0.63	0.88	1.55	0.90	0.46	TBR Choline L3	**0.76**	0.08	0.00	0.62	0.91	1.75	0.80	0.72
TBR Fluciclovine AORTA	**0.70**	0.07	0.00	0.57	0.83	2.90	1.00	0.59	SUV max Choline	**0.75**	0.07	0.00	0.61	0.89	5.45	0.70	0.74

**Table 7:** Univariate and multivariate logistic regression to predict malignant lesion (lesion-based analysis, *n =* 45/95).

Clinical and PET Parameters	UNIVARIATE		MULTIVARIATE 1		MULTIVARIATE 2	
	OR (95% CI)	*p* Value	OR (95% CI)	*p*-Value	OR (95% CI)	*p-*Value
iPSA	0.98 (0.87–1.03)	0.2	-	-	-	-
Clinic stage		0.6	-	-	-	-
cT1	1.0→(Ref)					
cT2	1.56 (0.35–6.91)					
Clinic ISUP			-	-	-	-
2	1.0→(Ref)	0.8				
3	1.84 (0.42–8.22)	0.4				
4	4.00 (0.25–63.95)	0.3				
5	1.89 (0.41–8.78)	0.4				
PIRADS v.2			-	-	-	-
1–2	1.0→(Ref)	0.2				
3	3.50 81.04–11.71)	0.04				
4	1.90 (0.27–4.55)	0.9				
5	1.40 (0.47–4.13)	0.5				
SUV max Choline PET/CT	1.65 (1.27–2.14)	**<0.001**	1.57 (0.93–2.63)	0.09	-	-
TBR aorta Choline PET/CT	2.36 (1.61–3.48)	**<0.001**	6.12 (1.51–24.79)	**0.01**	-	-
TBR L3 Choline PET/CT	2.60 (1.41–4.81)	**0.002**	0.2 (0.04–1.03)	*0.05*	-	-
TBR Liver Choline PET/CT	47.00 (5.85–377.63)	**<0.001**	0.02 (0.00–51.22)	0.3	-	-
SUV max Fluciclovine PET/CT	1.58 (1.22–2.04)	**0.001**	-	-	1.34 (0.75–2.37)	0.3
TBR aorta Fluciclovine PET/CT	1.81 (1.33–2.46)	**<0.001**	-	-	1.30 (0.81–2.10)	0.3
TBR L3 Fluciclovine PET/CT	4.77 (2.19–10.38)	**<0.001**	-	-	3.96 (0.93–16.87)	*0.06*
TBR Liver Fluciclovine PET/CT	32 (4.89–221.90)	**<0.001**	-	-	0.07 (0.00–19.72)	0.4

Legend: Target to Background Ratio (TBR); L3 (bone marrow background measured at L3 vertebral body); *p* < 0.05 was considered the threshold for statistical significance and reported in bold. *p* = 0.05 is reported in italics being close to the statistical significance.

## Data Availability

Data available on request due to privacy restrictions.

## Supplementary Materials

The following are available online at https://www.mdpi.com/article/10.3390/cancers13071575/s1, Figure S1: The workflow diagram of the study, Table S1: [^18^F]Fluciclovine and [^11^C]Choline PET uptake values (mean ± standard deviation; median; range) for each benign histo-type (*n =* 31).
