# Supplementary material for: The Role of [18F]Fluciclovine PET/CT in the Characterization of High-Risk Primary Prostate Cancer: Comparison with [11C]Choline PET/CT and Histopathological Analysis
Source: Cancers (Basel). 2021 Mar 29;13(7):1575. doi: 10.3390/cancers13071575 (PMC8037300; doi:10.3390/cancers13071575)
Supplement: Supplementary file 1 [file cancers-13-01575-s001.pdf]

cancers

MDPI

# The Role of [18F]Fluciclovine PET/CT in the Characterization of High-Risk Primary Prostate Cancer: Comparison with [11C]Choline PET/CT and Histopathological Analysis

**Lucia Zanoni, Riccardo Mei, Lorenzo Bianchi, Francesca Giunchi, Lorenzo Maltoni, Cristian Pultrone, Cristina Nanni, Irene Bossert, Antonella Matti, Riccardo Schiavina, Michelangelo Fiorentino, Cristina Fonti, Filippo Lodi, Antonietta D'Errico, Eugenio Brunocilla and Stefano Fanti**

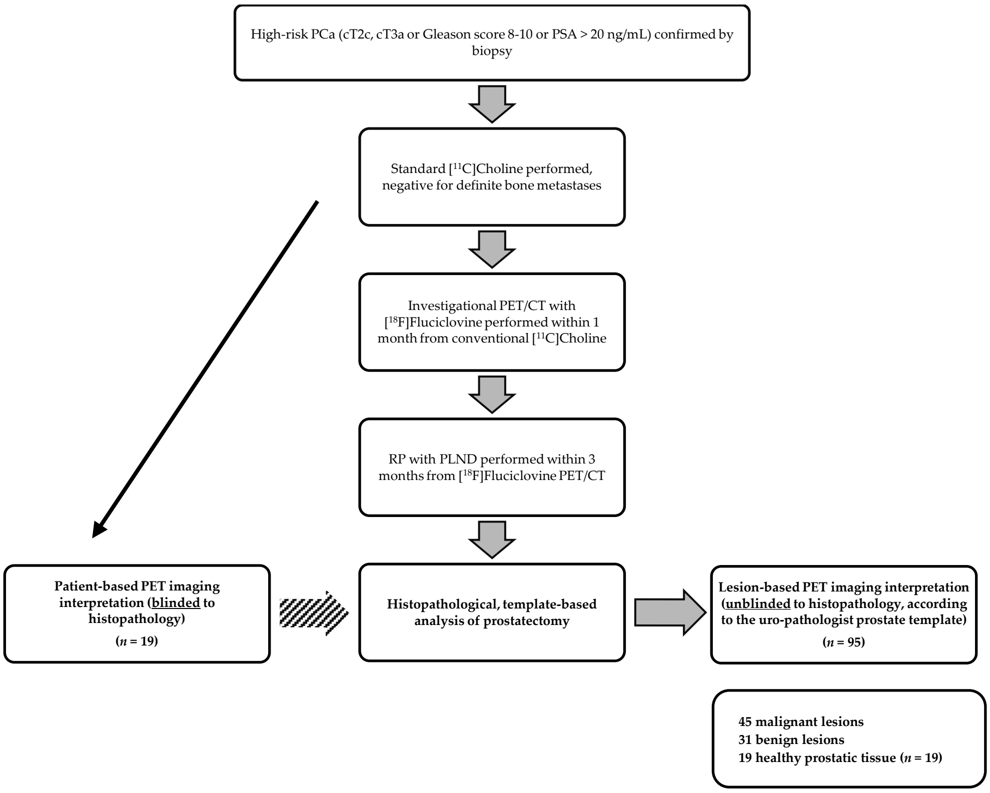


**Figure S1.** The workflow diagram of the study.

**Table S1.** [$^{18}$F]Fluciclovine and [$^{11}$C]Choline PET uptake values (mean±standard deviation; median; range) for each benign histo-type (*n* = 31).

| PET Semi-Quantitative Parameters. Values Expressed in: Mean ± Standard Deviation; Median (Range) | PIN (*n* = 6) | Inflammation (*n* = 16) | PHA (*n* = 6) | PAH+Inflammation (*n* = 2) | Infarction (*n* = 1) |
|---|---|---|---|---|---|
| SUV max Choline | 4.9 ± 0.8; 5.2 (3.5–5.6) | 4.0 ± 2.1; 4.3 (1.8–6.6) | 3.8 ± 0.7; 3.8 (3.1–4.9) | 5.0 ± 0.6; 5.0 (4.5; 5.4) | 6.3 |
| SUV max Fluciclovine | 4.4 ± 1.4; 5.1 (2.2–5.8) | 4.6 ± 2; 4.7 (2.8–8.4) | 5.0 ± 1; 4.8 (3.9–6.7) | 4.0 ± 0.9; 4.0 (3.4–4.6) | 4.5 |
| TBR Choline Prostate | 1.6 ± 0.3; 1.5 (1.3–2.2) | 1.3 ± 0.4; 1.4 (0.7–2.0) | 1.3 ± 0.3; 1.3 (0.8–1.5) | 2.2 ± 0.9; 2.2 (1.6–2.8) | 1.9 |
| TBR Fluciclovine Prostate | 1.3 ± 0.2; 1.1 (1.1–1.7) | 1.3 ± 0.5; 1.0 (0.8–2.5) | 1.3 ± 0.4;1.3 (0.8–1.9) | 1.3 ± 0.1; 1.3 (1.3–1.4) | 1.7 |
| TBR Choline AORTA | 3.1 ± 0.41;3.0 (2.7–3.9) | 2.7 ± 1.2; 2.4 (1.5–4.4) | 3.1 ± 0.6; 3.0 (2.4–3.7) | 4.4 ± 0.8; 4.4 (3.9; 5.0) | 4.5 |
| TBR Fluciclovine AORTA | 3.8 ± 0.9; 3.9 (2.3–4.7) | 3.1 ± 1.8; 3.5 (1.5–7.4) | 3.4 ± 0.6; 3.5 (2.2–3.9) | 3.2 ± 0.9; 3.2 (2.6–3.8) | 3.5 |
| TBR Choline L3 | 1.9 ± 0.3; 1.9 (1.6–2.5) | 1.3 ± 0.7; 1.4 (0.5–2.3) | 1.4 ± 0.3; 1.4 (0.9–1.8) | 2.6 ± 0.8; 2.6 (2.1–3.2) | 2.4 |
| TBR Fluciclovine L3 | 1.6 ± 0.4; 1.6 (1.1–2.0) | 1.2 ± 0.7; 1.6 (0.5–2.2) | 1.5 ± 0.2; 1.5 (1.3–1.8) | 1.7 ± 0.6; 1.7 (1.3–2.1) | 2.8 |
| TBR Choline LIVER | 0.6 ± 0.1; 0.6 (0.5–0.7) | 0.5 ± 0.2; 0.5 (0.2–0.8) | 0.5 ± 0.1; 0.5 (0.4–0.7) | 0.8 ± 0.1; 0.8 (0.7–0.8) | 0.8 |
| TBR Fluciclovine LIVER | 0.6 ± 0.1; 0.7 (0.4–0.7) | 0.6 ± 0.3; 0.6 (0.3–1.0) | 0.7 ± 0.1; 0.8 (0.5–0.9) | 0.7 ± 0.01; 0.7 (0.6–0.7) | 0.9 |

Legend: Target to Background Ratio (TBR); L3 (bone marrow background measured at L3 vertebral body); Interquartile range (IQR); Prostate Intraepithelial Neoplasia (PIN); post-atrophic hyperplasia (PAH).
